# Identification of High- and Low-Cadmium (Cd)-Accumulating Rice Cultivars Using Combined Molecular Markers

**DOI:** 10.3390/plants14182931

**Published:** 2025-09-20

**Authors:** Chengcheng Wang, Fangfang Ding, Qinlei Rong, Zhihong Lu, Junru Fu, Chunhuo Zhou

**Affiliations:** 1 College of Land Resources and Environment, Jiangxi Agricultural University, Nanchang 330045, China; 2 Key Laboratory of Agricultural Resources and Ecology in Poyang Lake Watershed of Ministry of Agriculture and Rural Affairs in China, Nanchang 330045, China; 3 College of Agriculture, Jiangxi Agricultural University, Nanchang 330045, China

**Keywords:** cadmium accumulation, rice cultivars, genotypic variation, marker-assisted selection, food safety

## Abstract

Rice grain is a primary dietary source of cadmium (Cd), a heavy metal toxic to humans. Reducing Cd accumulation in rice through selecting and breeding low-Cd-accumulating cultivars is very important. However, field-based screening for low-Cd rice cultivars remains labor-intensive and time-consuming. In this study, we identified molecular marker genotypes that can distinguish high- and low-Cd-accumulating rice cultivars. We developed corresponding genotypes for marker-assisted selection of low-Cd cultivars in both early and late rice varieties. Fifty-nine locally adapted, high-yielding early rice cultivars and thirty-seven locally adapted, high-yielding late rice cultivars were grown in two fields with different soil Cd levels and genotyped using molecular markers associated with grain Cd accumulation. We identified five early rice cultivars that consistently showed low Cd accumulation, with grain Cd concentrations below the food safety threshold of 0.2 mg kg^−1^ across two paddy fields. For early rice, we developed two low-Cd combined molecular marker genotypes (Multi-LCL1 and Multi-LCL2) that had significantly lower grain Cd content compared to Multi-LCL3 and Multi-LCL4. For late rice, the low-Cd combined molecular marker genotype Multi-CL1 showed substantially reduced grain Cd levels relative to Multi-CL2-CL5. These findings suggest that the combined molecular marker genotypes Multi-LCL1/LCL2 for early rice and Multi-CL1 for late rice are practical tools for quickly identifying cultivars with low Cd accumulation potential.

## 1. Introduction

Cadmium (Cd), a toxic heavy metal widely present in agricultural soils, has been classified as a Group 1 carcinogen by the International Agency for Research on Cancer (IARC) and the National Toxicology Program (NTP) [1,2]. Its environmental prevalence stems from both natural pedogenic processes, such as mineral weathering, and anthropogenic activities, including mining, waste disposal, and application of fertilizers and agrochemicals [3,4]. A nationwide soil survey in China revealed that 7% of the sampled soils were contaminated with Cd [5]. Furthermore, studies have documented a significant increase in soil Cd concentrations across multiple regions in southern China over the past four decades [6,7,8].

Rice, a staple food for nearly half of the global population, serves as the primary dietary source of Cd for populations dependent on rice [9,10,11]. Long-term exposure to elevated Cd levels can lead to severe health issues, including renal dysfunction and Itai-Itai disease [12,13,14]. Dietary exposure assessments in China indicate that rice contributes to 56% of the total Cd intake in the general population [15]. Notably, certain population groups in several Asian countries, particularly infants and children, are exposed to Cd at levels exceeding the tolerable intake limit established by the FAO/WHO [8,16,17,18]. Compared to other heavy metals, Cd exhibits a higher transfer efficiency from soil to the edible parts of crops [10]. Moreover, rice demonstrates a higher capacity for Cd uptake than wheat and other major cereal grains [15,19,20]. As the world’s largest rice producer, China relies on rice as a staple food for the majority of its population, especially in the southern regions [21]. Therefore, reducing the transfer of Cd from soil to rice grain is an urgent priority for ensuring food safety and safeguarding public health.

Several factors contribute to elevated Cd levels in rice grains in southern China, including soil contamination, irrigation water quality, soil acidification, and the cultivation of high-Cd-accumulating rice cultivars [6,22]. To mitigate Cd accumulation in grains, various strategies can be employed depending on soil conditions and contaminant properties, such as liming acidic soils, optimizing water management in paddies, and selecting or breeding low-Cd-accumulating cultivars [6,23]. Several recent reviews have comprehensively summarized advances in understanding Cd uptake, transport, and genetic regulation in rice, as well as various agronomic and breeding strategies for mitigation [11,24,25]. Liming has proven effective in reducing Cd bioavailability in acidic soils, thereby decreasing plant uptake [22]. Water management also significantly influences Cd accumulation; specifically, maintaining flooded conditions has been shown to reduce Cd levels in rice grains [10,26,27]. Recent studies indicate that phosphorus and silicon fertilizers can alleviate Cd toxicity and reduce its accumulation in cereal crops [28,29]. In a novel approach, Zhang et al. employed automated machine learning models to predict heavy metal concentrations in crop grains, using ten variables—including organic fertilizer application rate, heavy metal concentration in organic fertilizers, soil heavy metal concentration, organic matter content, pH, cation exchange capacity, clay, sand, silt content, and crop type—as inputs, with grain Cd concentration as the output [30].

Breeding rice cultivars with inherently low Cd accumulation represents a practical and feasible strategy. Substantial genetic variation exists among rice cultivars in their ability to accumulate Cd in grains, with *indica* types generally exhibiting higher grain Cd concentrations than *japonica* types [6,31,32]. For example, a 40-fold variation in grain Cd concentration was reported among 1763 rice accessions from diverse geographical origins [33]. Similarly, field trials on moderately contaminated soils in South China demonstrated a 10- to 32-fold range in brown rice Cd among 471 locally adapted and high-yielding rice cultivars [31]. Tang et al. further observed variations of 21-, 16- and 22-fold in brown rice Cd across different environments among 167 high-yielding cultivars [32]. These findings demonstrate the considerable heritable genetic variability in rice germplasm, which can be exploited to reduce grain Cd accumulation. Consequently, screening locally adapted high-yielding cultivars for stable low-Cd accumulation is a practical and effective approach to reduce grain Cd content without compromising yield.

Understanding the transfer of Cd from soil to the food chain is critical for food safety. Numerous key genes involved in Cd uptake, translocation, sequestration, and detoxification in rice have been identified and characterized [10,24,25]. Although allelic variations in some genes contributing to differential Cd accumulation are known, the underlying molecular mechanisms remain incompletely understood. Insights into the molecular mechanisms of Cd uptake and translocation offer potential targets for marker-assisted breeding of low-Cd rice cultivars. Furthermore, multiple quantitative trait loci (QTLs) controlling grain Cd concentrations have been identified [34,35], and the combination of several QTLs associated with low accumulation traits through marker-assisted breeding programs holds promise [10]. On this basis, several studies have proposed using genetic engineering or marker-assisted selection (MAS) to reduce Cd accumulation while simultaneously increasing essential trace element contents, thereby improving the nutritional quality and safety of crops [36].

However, despite known genetic variation, the practical application of MAS for developing low-Cd rice is often hindered by the moderate accuracy of single markers and significant genotype-by-environment interactions [32,37]. Moreover, most studies focus on either a single rice season or a limited set of genetic materials, highlighting a need for a robust multi-marker system applicable across different seasons (early and late rice) and among diverse, locally adapted high-yielding cultivars.

To address this gap, this study aimed to develop and validate combined molecular markers for the rapid and accurate identification of low- and high-Cd-accumulating cultivars in both early and late rice seasons. We screened a panel of 59 locally adapted high-yielding early cultivars and 37 late cultivars, grown in two paddy fields with varying soil Cd concentrations. Genotyping was performed using molecular markers associated with three Cd-related QTLs for early rice and two for the late rice. Based on these genotypic data, we evaluated the efficacy of these markers in classifying cultivars into high- and low-Cd-accumulating groups according to grain Cd content.

## 2. Results

### 2.1. Total Cadmium Concentration in the Soils

Field trials were conducted on early and late rice cultivars across four field sites (Figure S1). All soils were acidic. Field A had a moderate Cd concentration of 0.48 mg kg^−1^, which exceeded the limit stipulated by China’s Soil Environmental Quality Standard (GB 15618-2018) for acidic soils (0.3 mg kg^−1^ at pH < 5.5; 0.4 mg kg^−1^ at pH 5.5–6.5) (Table 1). Fields B, C, and D exhibited higher levels of Cd contamination, ranging from 0.6 to 0.8 mg kg^−1^ (Table 1). In 2024, a total of 59 widely cultivated early rice cultivars were grown at sites in Yichun and Shangrao, while 37 late-season cultivars were evaluated in Yichun and Ganzhou.

### 2.2. Cadmium Concentrations in Rice Grain

**Figure 1:**
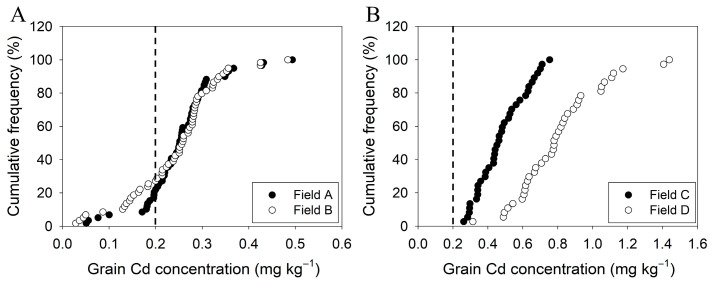
Cumulative frequency of Cd concentration in brown rice harvested from two sites of early rice cultivars (**A**) and two sites of late rice cultivars (**B**). The vertical dash line indicates Chinese Food Hygiene Standards for rice Cd concentration (0.2 mg kg^−1^).

Among the 59 early rice cultivars tested, grain Cd concentrations ranged from 0.05 to 0.49 mg kg^−1^ in Field A and from 0.03 to 0.48 mg kg^−1^ in Field B, representing 10- and 17-fold variation among cultivars, respectively (Figure 1A). Approximately 78% (Field A) and 73% (Field B) of these cultivars exceeded the maximum permissible level of Cd in rice (0.2 mg kg^−1^) specified in China’s National Food Safety Standard (GB 2762-2022). Cultivars with grain Cd concentrations < 0.2 mg kg^−1^ were classified as low-Cd accumulators, while the rest were classified as high-Cd accumulators. Those that consistently maintained grain Cd concentrations below 0.2 mg kg^−1^ in both fields were designated as stably low-Cd accumulators, five such early rice cultivars identified (Table S2).

Among the 37 late rice cultivars tested, grain Cd concentrations ranged from 0.26 to 0.75 mg kg^−1^ in Field C and from 0.31 to 1.44 mg kg^−1^ in Field D, showing 3-fold and 5-fold variation among cultivars, respectively (Figure 1B). All late rice cultivars (100%) in both fields exceeded the 0.2 mg kg^−1^ limit. Consequently, no late rice cultivars qualified as stably low-Cd accumulators.

### 2.3. Screening of Rice Genotypes for High- and Low-Cd-Accumulating Cultivars Using Molecular Markers

Among 13 markers screened, three (LCd41, CAL1, LCd-38) were polymorphic in 59 early rice cultivars, and two (CAL1, LCd31) exhibited polymorphism in the 37 late rice cultivars used in this study (Table S1 and Figure S2). These polymorphic markers were thus used to genotype their respective cultivar panels.

**Early rice:** Genotyping results showed that markers LCd-41, CAL1, and LCd-38 classified 3.4%, 1.7%, and 22.0% of the 59 early rice cultivars as low-Cd genotypes, respectively (Table 2). The accuracy of identifying high- and low-Cd accumulators was evaluated by comparing the marker-based genotypic predictions with actual grain Cd concentrations of cultivars grown in two distinct field sites. A threshold of 0.2 mg kg^−1^ (consistent with GB 2762-2022) was used to distinguish high- and low-Cd phenotypes during field screening. Overall, the three individual molecular markers performed well in predicting the high-Cd grain phenotype across both fields, with prediction accuracies ranging from 74% to 87% (Table 2). In contrast, their accuracy in predicting the low-Cd phenotype was lower, varying from 38% to 100% (Table 2). A trade-off was observed between the prediction accuracy of high- and low-Cd phenotypes. The overall prediction accuracy-defined as the proportion of cultivars correctly classified (i.e., high-Cd genotype with high-Cd phenotype, or low-Cd genotype matched with low-Cd phenotype) among the 59 early rice cultivars-ranged as follows: 76–81% (average 78%) for marker LCd-41; 74–79% (average 77%) for CAL1; and 67–79% (average 73%) for LCd-38 (Table 2).

**Late rice:** Markers CAL1 and LCd-31 identified 2.7% and 91.9% of the 37 late rice cultivars as low-Cd genotypes, respectively (Table 3). Both markers predicted high-Cd phenotypes with 100% accuracy but failed to identify any low-Cd phenotypes (0% accuracy). Across two field trials, the overall accuracy was 97% for CAL1 but only 8.1% for LCd-31 (Table 3).

Through comparative analysis of genetic diversity and grain phenotypes across different field trials, we found that the combined molecular marker panels (LCd-41-CAL1-LCd-38 for early rice and CAL1-LCd-31 for late rice) achieved the highest accuracy in identifying high- and low-Cd accumulators among all tested markers (Table 2 and Table 3). Additionally, the mean prediction accuracy of the LCd-41-CAL1-LCd-38 panel and CAL1-LCd-31 panel ranked the highest for early and late rice cultivars, respectively (Table 2 and Table 3).

For the combined molecular marker panels, two-way ANOVA was performed on pooled data from different fields to analyze the sources of variation in grain Cd concentrations. The results showed that grain Cd concentrations were significantly affected by genotype, field, and genotype × field interactions (Table 4). For early rice cultivars, the marker panel, field, and marker panel × field interaction accounted for 13%, 84.4%, and 84.7% of the total phenotypic variation in grain Cd concentration, respectively; the corresponding values for late rice cultivars were 9.1%, 43.7%, and 46.7% (Table 4).

### 2.4. Efficacy of Combined Molecular Markers in Classifying High- and Low-Cd-Accumulating Rice Cultivars

To assess the ability of different combined molecular marker panels to differentiate rice cultivars based on grain Cd levels, a comprehensive analysis was conducted using genotype data and grain Cd concentrations from multi-environment field trials. Comparative analysis of genotypes and grain Cd concentrations revealed significant genetic diversity within the combined molecular marker panels for both early and late rice cultivars. Specifically, four distinct genotypes were identified among the early rice cultivars, and five among the late rice cultivars, these genotypes effectively distinguished between high- and low-Cd-accumulating cultivars (Table S3). The distribution of these marker combinations across different rice cultivars was highly diverse, indicating inherent genetic variations underlying Cd accumulation traits.

**Figure 2:**
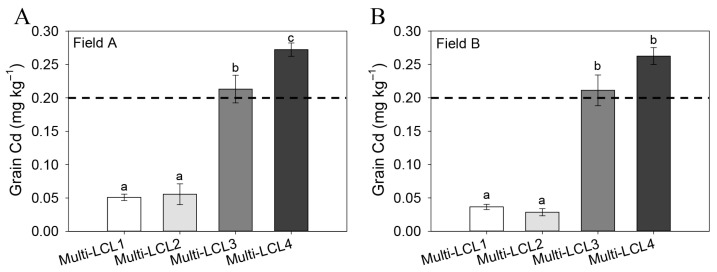
Analysis of grain cadmium concentration in early rice cultivars (**A**,**B**) using the combined molecular markers. Plants were grown in two Cd-polluted paddy sites (Field A and B) located in Jiangxi province, China. The horizontal dashed line indicates Chinese Food Hygiene Standards for rice Cd concentration (0.2 mg kg^−1^). Data are presented as mean ± SE (n = 3 plots per genotype per field). Bars within the same field site (A or B) marked with different letters are significantly different based on one-way ANOVA followed by Tukey’s test (*p* < 0.05).

**Figure 3:**
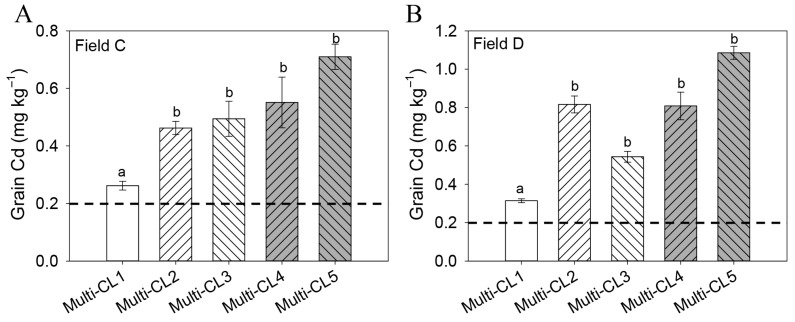
Analysis of grain cadmium concentration in late rice cultivars (**A**,**B**) using the combined molecular markers. Plants were grown in two Cd-polluted paddy sites (Field C and D) located in Jiangxi province, China. The horizontal dashed line indicates Chinese Food Hygiene Standards for rice Cd concentration (0.2 mg kg^−1^). Data are presented as mean ± SE (n = 3 plots per genotype per field). Bars within the same field site (C or D) marked with different letters are significantly different based on one-way ANOVA followed by Tukey’s test (*p* < 0.05).

In early rice cultivars, the marker-based genotypes multi-LCL1 and multi-LCL2 exhibited stably low Cd accumulation across Field A and Field B, while multi-LCL3 and multi-LCL4 showed stably high Cd accumulation (Figure 2A,B). This consistent association between genotype and Cd accumulation phenotype suggests a strong genetic basis for rice grain Cd accumulation. For late rice cultivars, the combined marker-based genotype multi-CL1 consistently displayed significantly lower grain Cd concentrations across Field C and Field D compared to other marker combinations (Figure 3A,B), indicating its potential to identify cultivars with the lowest grain Cd levels.

Overall, the combined molecular marker panels for both early and late rice cultivars demonstrated promising applicability in classifying cultivars by their grain Cd accumulation capacity. Thus, the marker combinations corresponding to multi-LCL1 and multi-LCL2 in early rice, and multi-CL1 in late rice, are particularly valuable for identifying low-Cd-accumulating cultivars, while other marker combinations can effectively distinguish high-Cd-accumulating cultivars. These findings highlight the significant potential of these combined molecular marker panels for application in breeding programs aimed at reducing Cd accumulation in rice grains.

## 3. Discussion

Substantial genotypic variation in rice grain Cd concentration has been well-documented in numerous studies, reflecting the complex interplay between genetic background and environmental factors [31,32,33,37,38,39]. In this study, we observed 10–17-fold variation in grain Cd among 59 early rice cultivars and 3–5-fold variation among 37 late rice cultivars, all of which are high-yielding and widely cultivated in Jiangxi and neighboring provinces. Notably, five early rice cultivars consistently accumulated grain Cd below China’s maximum permissible limit (0.2 mg kg^−1^, GB 2762-2022) across both experimental fields (Table S2). These cultivars represent promising genetic resources for immediate deployment in slightly to moderately Cd-contaminated paddies in South China. Multi-location yield validation is recommended before any low-Cd line is advanced to commercial release. However, the stability of these low-Cd phenotypes must be validated through multi-environment trials over consecutive growing seasons, especially given the significant genotype-by-environment (G×E) interactions observed in Cd accumulation [31,37]. Since the implementation of the Action Plan on Soil Pollution Prevention and Control (‘Soil Action Plan’) in 2016 [40], local governments have encouraged farmers to apply lime to soils and cultivate rice varieties with low Cd accumulation potential. However, all late cultivars exceeded the grain Cd limit in this study. The significantly lower Cd concentrations in early versus late cultivars likely reflect both genotypic differences and reduced Cd bioavailability due to the wetter growing conditions in the early season. This pronounced difference underscores the need for season-specific breeding strategies (Figure 1A,B).

The genetic basis of Cd accumulation in rice is governed by multiple quantitative trait loci (QTLs) and key genes involved in Cd uptake, translocation, and sequestration [39,41,42,43,44]. Recently, numerous molecular markers associated with QTLs controlling Cd accumulation in rice grains have been identified and applied in marker-assisted breeding [43,45,46,47]. In this study, we focused on three markers (LCd-41, CAL1, LCd-38) for early rice and two (CAL1, LCd-31) for late rice, all located within or near known Cd-related QTLs. The CAL1 gene, for instance, encodes a defensin-like protein that facilitates Cd efflux and allocation in rice [48,49]. The accuracy for identifying late cultivars with high grain Cd concentrations exceeded that for low-Cd types, presumably because Cd accumulation is a more stable trait in Cd-contaminated paddy soils [37,46,50].

Grain Cd accumulation is governed by multiple genetic loci and genotype × environment interactions, particularly involving factors influencing Cd bioavailability (Table 4) [31,32,37,50]. Among the markers tested, the combination LCd-41–CAL1–LCd-38 showed the highest overall accuracy for early rice (Table 2), while CAL1–LCd-31 performed best for late rice (Table 3). The superior performance of the combined marker sets (LCd-41-CAL1-LCd-38 for early rice and CAL1-LCd-31 for late rice) suggests that these loci may act synergistically to regulate Cd accumulation. This multi-locus approach mitigates the limitations of single-marker selection, which is often compromised by epistatic interactions and environmental variability [32,37]. A relatively high proportion of total SSE/SST was observed for each field in this study (Table 4). The large contribution of field conditions to the total variation in grain Cd accumulation for combined markers was likely attributable to greater differences in climatic conditions and soil types between the two fields for early and late rice cultivars.

Field trials confirmed that early rice lines carrying the Multi-LCL1 and Multi-LCL2 combined marker genotypes exhibited significantly lower grain Cd concentrations than those with Multi-LCL3 and Multi-LCL4 (Figure 2A,B). Critically, grain Cd levels in both Multi-LCL1 and Multi-LCL2 lines consistently remained below the safety threshold (<0.2 mg/kg) (Figure 2A,B). These marker genotypes are thus valuable for early pre-screening and marker-assisted breeding of low-Cd early rice varieties. We recommend using lines with Multi-LCL1 or Multi-LCL2 genotypes as parental material in breeding programs targeting reduced grain Cd.

Similarly, late rice lines with the Multi-CL1 combined marker genotype showed significantly lower grain Cd content than those carrying Multi-CL3, Multi-CL4, or Multi-CL5 across field sites (Figure 3A,B). This identifies Multi-CL1 as a low-Cd combined marker genotype suitable for selection in late rice breeding programs, supporting the development of low-Cd late cultivars through MAS. For efficient and stable reduction of Cd accumulation, late rice lines harboring the Multi-CL1 genotype are recommended as breeding parents.

Our results demonstrate that the combined marker genotypes Multi-LCL1 and Multi-LCL2 in early rice, and Multi-CL1 in late rice, are robust predictors of low Cd accumulation across contrasting soil Cd conditions. The consistently low grain Cd levels in these genotypes—even in soils exceeding national safety standards—highlight their potential for MAS. Nevertheless, the low frequency of these favorable genotypes (e.g., only two early rice cultivars with Multi-LCL1/LCL2 and one late rice cultivar with Multi-CL1) indicates that current elite germplasm in Cd-affected regions lacks diversity in low-Cd alleles. This scarcity underscores the urgency of introgressing these alleles into high-yielding backgrounds through strategic breeding programs.

Contrary to some reports suggesting hybrid rice may accumulate more Cd [51], we found no consistent difference between hybrid and inbred cultivars across most fields (Figure S3), supporting the view that genetic diversity—rather than breeding type—is the primary determinant of Cd accumulation [52]. This aligns with recent studies indicating that Cd accumulation is a polygenic trait influenced by numerous small-effect loci and their interactions with the environment [32,34,46]. It is important to note that the dCAPS markers employed in this study (e.g., LCd-41, LCd-38) are optimized for distinguishing homozygous genotypes. While they proved highly effective for screening the inbred lines utilized here, their resolution may be insufficient for reliably discriminating heterozygous loci in hybrid varieties, owing to the small differences in restriction fragment sizes resolved on agarose gels. For applications involving hybrid populations, we therefore recommend employing higher-resolution genotyping methods, such as capillary electrophoresis or Kompetitive Allele-Specific PCR (KASP) assays. Moving forward, integrating high-throughput genotyping platforms (e.g., KASP assays) with machine learning models—as recently proposed by Zhang et al. [30]—could further enhance the predictive accuracy and scalability of MAS for low-Cd rice breeding.

## 4. Materials and Methods

### 4.1. Rice Cultivars

A total of 59 locally adapted, high-yielding early rice cultivars were cultivated at two field sites containing moderate levels of Cd from April to July 2024. Additionally, 37 locally adapted, high-yielding late rice cultivars were grown at two field sites with moderate levels of Cd from July to October 2024. These cultivars were collected from Jiangxi and neighboring provinces and were commonly cultivated over large areas. All early cultivars were *Indica* rice, with 38 being hybrid varieties and 21 being conventional varieties. Among the late cultivars, which were all *India* rice as well, 31 were hybrid varieties and the remaining 6 were conventional.

### 4.2. Field Sites

The two paddy fields for early rice cultivars (field A and B) were located in Yichun City and Shangrao City, Jiangxi Province, China, respectively (Figure S1). The two paddy fields for late rice cultivars (field C and D) were located in Yichun City and Ganzhou City, Jiangxi Province, respectively (Figure S1). Total Cd concentrations in the paddy soils ranged from 0.4 to 0.8 mg kg^−1^ in Yichun, Shangrao and Ganzhou (Table 1), exceeding the Chinese national standard for soil environmental quality (GB 15618, 2018). This standard sets Cd limits at 0.3 mg kg^−1^ for soils with pH < 5.5 and 0.4 mg kg^−1^ for soils with pH < 6.5. The soils at the Yichun, Shangrao and Ganzhou sites are acidic (pH 5.13–5.76). Specifically, the mean soil Cd concentrations and pH values were: field A (0.48 mg kg^−1^, pH 5.13), field B (0.67 mg kg^−1^, pH 5.61), field C (0.78 mg kg^−1^, pH 5.45), and field D (0.69 mg kg^−1^, pH 5.76).

### 4.3. Rice Cultivation

Seeds of early rice cultivars were germinated and sown on seedbeds in March 2024. Seedlings were transplanted into fields A and B in April 2024 using a randomized block design, with three replicates per field. Seeds of late rice cultivars were germinated and sown on seedbeds in June 2024, and their seedlings were transplanted into fields C and D in July 2024, also following a randomized block design with three replicates per field. Within each replicate, cultivars were planted in two rows of ten hills per row, with one seedling per hill and 20 cm spacing between rows and between hills within rows. Paddy field water management followed local practices: fields were flooded throughout the rice growth season but drained during the late tillering stage and for 10 days prior to final harvest. Fertilizers, fungicides, and pesticides were applied according to local recommendations for rice production. Plants of each cultivar were harvested 30 days after full heading. Grain from the central eight hills of each plot was harvested and pooled for elemental analysis.

### 4.4. Genomic DNA Isolation and Genotyping

Thirteen molecular markers associated with Cd accumulation in rice grains were initially selected from public references and patents. Preliminary screening identified three polymorphic markers (LCd-41, LCd-38, and CAL1) in early rice cultivars and two polymorphic markers (CAL1 and LCd-31) in late rice cultivars (Table S1). All markers were single nucleotide polymorphisms (SNPs) [53,54,55,56]. Derived cleaved amplified polymorphic sequence (dCAPS) markers were designed for LCd-41, LCd-38, and LCd-31 using dCAPS Finder 2.0 [57]. Genomic DNA was extracted from leaves of each cultivar using the cetyl trimethyl ammonium bromide (CTAB) mini-prep method [58]. PCR amplification was performed for all four markers using the primer pairs listed in Table S1. The PCR products for LCd-41, LCd-38, and LCd-31 were digested with appropriate restriction endonucleases in a 10 μL reaction volume according to the manufacturer’s instructions. Both the digested products (for LCd-41, LCd-38, LCd-31) and the undigested PCR products were separated by electrophoresis on 1.5–4.0% agarose gels. Gels were stained with ethidium bromide and visualized using a BIO-RADXR gel imaging analysis system (Bio-Rad, Hercules, CA, USA). The CAL1 SNP in rice cultivars was genotyped by sequencing on an Illumina HiSeq platform (PE150) performed by Wuhan GenoSafe Biotechnology Co., Ltd. (Wuhan, China).

### 4.5. Rice Grain and Soil Analysis

Soil samples were collected from the plow layer (0–20 cm depth) of each field site prior to transplanting. Samples were air-dried, crushed, and sieved through a 2-mm nylon mesh. A subsample of each sieved soil was then ground with an agate grinder to pass through a 0.15-mm nylon sieve. Soil pH was measured potentiometrically using a combined glass electrode in a soil-deionized water suspension (1:2.5 *w*/*v*; <2 mm fraction). For soil Cd analysis, finely ground soil samples (<0.15 mm) were digested with aqua regia (HCl:HNO_3_, 4:1 *v*/*v*) using a heating block digester, following McGrath and Cunliffe [59]. Rice grain samples were dried, dehusked, ground to a fine powder, and digested with HNO_3_/HClO_4_ (85:15 *v*/*v*; 5 mL) in a heating block. Cadmium concentrations in all digested solutions (soil and grain) were determined by inductively coupled plasma mass spectrometry (ICP-MS; Perkin-Elmer Nexion 300×, Shelton, CT, USA).

### 4.6. Statistical Analysis

All data were analyzed using two-way analysis of variance (ANOVA), followed by Student-Newman-Keuls multiple comparison tests, employing SPSS 27.0 software. For comparisons of grain Cd concentration among different combined marker genotypes within each field site, one-way analysis of variance (ANOVA) was performed, followed by Tukey’s test for multiple comparisons.

## 5. Conclusions

This study provides a validated molecular toolkit for rapidly identifying low-Cd rice cultivars using combined marker genotypes. We evaluated 59 early rice and 37 late rice cultivars across multiple field sites and identified five early rice cultivars with consistently low grain Cd accumulation below the national safety standard. The combined marker genotypes Multi-LCL1 and Multi-LCL2 effectively identified low-Cd early rice varieties, while Multi-CL1 selected late rice cultivars with relatively lower Cd content. These marker combinations offer a practical solution for reducing dietary Cd exposure without compromising yield and significantly decrease reliance on extensive field screening. Future work should focus on functional validation of the candidate genes underlying these markers, elucidating their roles in Cd transport and homeostasis, and expanding the breeding pool through allele mining and gene editing to develop climate-resilient, low-Cd rice varieties for Cd-contaminated regions.

## Figures and Tables

**Table 1:** Cadmium concentration in soils at the experimental sites.

Site	Cd (mg kg^−1^)
Field A	0.48
Field B	0.67
Field C	0.78
Field D	0.69

**Table 2:** Accuracy for identifying high- and low-Cd-accumulating early rice cultivars using three individual molecular markers and their combination.

	Genotype NO.		Field A			Field B			Mean Accuracy (%)
Marker	High-Cd Genotype	Low-Cd Genotype	High-Cd Accuracy (%)	Low-Cd Accuracy (%)	Overall Accuracy (%)	High-Cd Accuracy (%)	Low-Cd Accuracy (%)	Overall Accuracy (%)	
LCd-41	57	2	80.7	100.0	81.4	75.4	100.0	76.3	78.8
CAL1	58	1	79.3	100.0	79.7	74.1	100.0	74.6	77.1
LCd-38	46	13	87.0	53.8	79.7	76.1	38.5	67.8	73.7
LCd-41-CAL1-LCd-38	57	2	80.7	100.0	81.4	75.4	100.0	76.3	78.8

**Table 3:** Accuracy for identifying high- and low-Cd-accumulating late rice cultivars using two individual molecular markers and their combination.

	Genotype NO.		Field C			Field D			Mean Accuracy (%)
Marker	High-Cd Genotype	Low-Cd Genotype	High-Cd Accuracy (%)	Low-Cd Accuracy (%)	Overall Accuracy (%)	High-Cd Accuracy (%)	Low-Cd Accuracy (%)	Overall Accuracy (%)	
CAL1	36	1	100.0	0.0	97.2	100.0	0.0	97.2	97.2
LCd-31	3	34	100.0	0.0	8.1	100.0	0.0	8.1	8.1
CAL1-LCd-31	36	1	100.0	0.0	97.20	100.0	0.0	97.2	97.2

**Table 4:** Two-way ANOVA of the combined molecular markers for identifying high- and low-Cd-accumulating early and late rice cultivars.

	Variation	SS	df	MS	F	*p*-Value	SSE/SST (%) ^a^
Early rice cultivars	Markers	0.593	1	0.593	25.858	<0.001	13
	Field	21.41	2	10.705	466.425	<0.001	84.4
	Marker × Field	22.003	3	7.334	319.569	<0.001	84.7
	Error	3.971	173	0.023			
Late rice cultivars	Markers	0.264	1	0.264	7.143	0.009	9.1
	Field	2.038	1	2.038	55.177	<0.001	43.7
	Marker × Field	2.302	2	1.151	31.16	<0.001	46.7
	Error	2.622	71	0.037			

^a^ Sum of squares (ss) of each effect by total SS.

## Data Availability

The original data presented in this study are included in the article and its Supplementary Materials. Further inquiries can be directed to the corresponding authors.

## Supplementary Materials

The following supporting information can be downloaded at: https://www.mdpi.com/article/10.3390/plants14182931/s1. Table S1: Four molecular markers related with rice grain Cd accumulation; Figure S1: Location of the four field sites (A–D); Table S2: Stable low-Cd accumulating early rice cultivars selected based on two field trials; Table S3: Genotypes of combined molecular markers for identifying high- and low-Cd accumulating early and late rice cultivars. Figure S2: Polymorphism of the three molecular markers between high- and low-Cd accumulating rice cultivars (A: LCd-41; B: LCd-38; C: LCd31). Figure S3: Boxplots of grain Cd concentrations of early (A) and late (B) rice cultivars grouped into hybrid and non-hybrid. Table S4: Performance of candidate control cultivars ‘Zhong’an 2’ and ‘Shaoxiang 100’, demonstrating their unsuitability as stable positive controls due to environmentally variable Cd accumulation and mismatched genotypes.
