# Supplementary material for: Identification of High- and Low-Cadmium (Cd)-Accumulating Rice Cultivars Using Combined Molecular Markers
Source: Plants (Basel). 2025 Sep 20;14(18):2931. doi: 10.3390/plants14182931 (PMC12473944; doi:10.3390/plants14182931)
Supplement: Supplementary file 1 [file plants-14-02931-s001.zip › plants-3800315-supplementary.pdf]

Supplemental materials

Identification of high and low cadmium (Cd) accumulating rice cultivars using combined molecular markers

Chengcheng Wang[1,2], Fangfang Ding[1,2], Qinlei Rong[1,2], Zhihong Lu[1,2], Junru Fu[3], and Chunhuo Zhou[1,2,*]

[1] College of Land Resources and Environment, Jiangxi Agricultural University, Nanchang, China

[2] Key Laboratory of Agricultural Resources and Ecology in Poyang Lake Watershed of Ministry of Agriculture and Rural Affairs in China, Ministry of Agriculture and Rural Affairs, Nanchang, China

[3] College of Agriculture, Jiangxi Agricultural University, Nanchang, China

[*] Correspondence Author: Chunhuo Zhou;

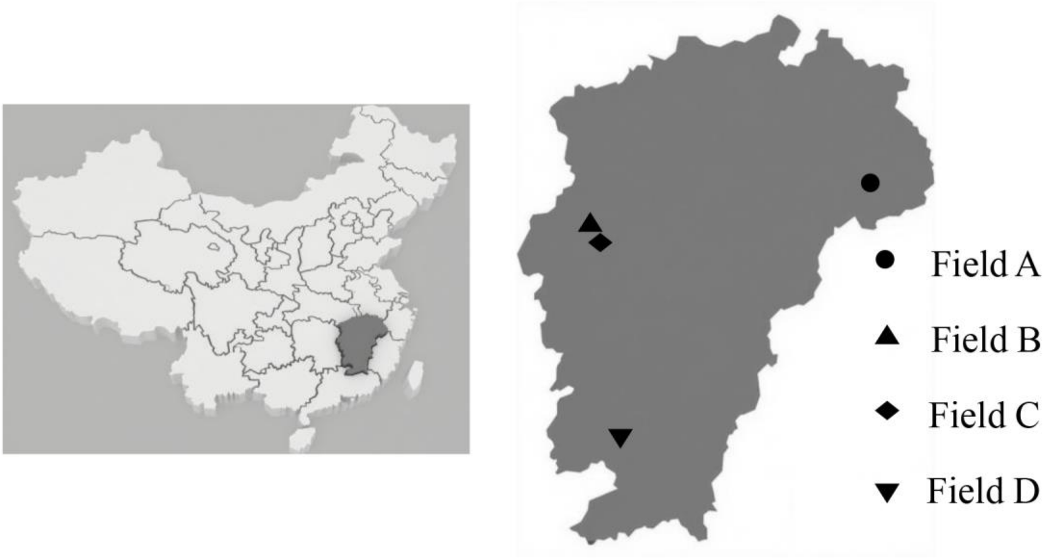

Figure S1. Location of the four field sites (A, B, C and D).

Table S1. Four molecular markers related with rice grain Cd accumulation

Marker	Primer	CHR	Sequence	Start position	Patent
LCd-41	F: GATGTCTCGCATCGATCAACCGGTTCC GCCAT<br>R: GACACTTCACATTCTCACAAAC	Chr10	GGCTCTCAACTCAAGTATTCATCCTTCTGAAATCTGATTTCTCTCTTTCCAATTACCAGTTTTTTTTTTTT<br>TTACTTTCCTCCTGAATAATCTCACTGCTTTAAATTCCTGATATCTTATGATGAAACTGCAAAATGTAAA<br>TTCAGACACAGCACAAGGTCGAGATGGACAAGAGGGACGGCAAGTTTGGACCTATGACAGTGCCTCC<br>AATGCAGCAGATGTCTCGCATCGATCAACCGGTTCCGCCTT**W**TGTTGGGTATGCGCCACAGGCACAGC<br>CAGCATACTACAGGTAGCCTGATGGTGGCTATCCAAGAGTTCAACATACAGATCATGCATGCTCAAGA<br>TTTTCTGTAGCAAAGTGAAGGATGGTGATCAAGTAGATACATAGTGTTTGTGAGAATGTGAAGTGTC	20844568	CN105671164B<br>(He et al., 2019)
LCd-38	F: GTCACAAAGAAGAAATAATGGC<br>R: TCTGCAACTCGTGTTTGTCTG	Chr10	GTCACAAAGAAGAAATAATGGCACCATGGTAACTATGCAAGTGGTGAAAGTCTTCAACTGAAAAACC<br>ATTTGCTTTTGTTTTCCTTATGGTGCAGTATTCTAACCAAATCAAGAATCATATGTAGAGATTGCAGGG<br>TATTTTATATTTTAATGAAACATTTCACAGGATAAGCATACAGATGCCTTCTCCAATACTGGCTTCAGA<br>CAAAAGCAAAGGATATCAGCTAAGACAGAATGATGATGTCATTGGCAGGCAGCAGGGAAGTGGTAAA<br>AGCACCCACA**Y**TGTGTAGGTAAGCATTCCATAAG	20089673	CN105543397B<br>(He et al., 2018)
LCd-31	F: GCGATAATTGCAATGAGTGTCTTGA<br>R: CCTTTGCTTGAAAAAAACTGGG	Chr9	CTAGTGGCGATAATTGCAATGAGTGTCTTTA**Y**CCATACAAATATGCATCATGACTCGATAGAAAACGGGA<br>GGATGTACATGGGAGTGCAGTTCTTTGGTACGTTGGCAATAATGTTTAAAGGGTTGGCAGAAATGGGAG<br>CAGCTCTTGCAAATCTCCCAGTTTTTTTCAAGCAAAGG	20089673	CN105624319B<br>(He et al., 2018)
CAL1	F: ACCAAGGTGGCGGAGGC<br>R: AGCCGACGAGCAATGCG	Chr2	CTGCCTGTCGCAGAGCCACAGGTTCAAGGGCATGTGCGTGAGCAGCAACAACTGCGCCAACGTGTGCA<br>GGACGGAGAGCTTCCCCGACGGCGAGTGCAAGTCGCACGGC**R**TCGAGCGCAAGTGCTTCTGCAAGAA<br>GGTCTGCTAGTGCATGCTAGCCCCGCTGTCTCTGCAGTCGCATTGCTCGTCGGCTGTGTATCTGCAGAG<br>ATTGTAGTCGCGTGTTCTCCTTTGATACTACAGGTAGCCTGATGGTGGCTATCCAAGAGTTCAACATAC<br>AGATCA	25190881	CN108250280A<br>(Gong et al., 2018)

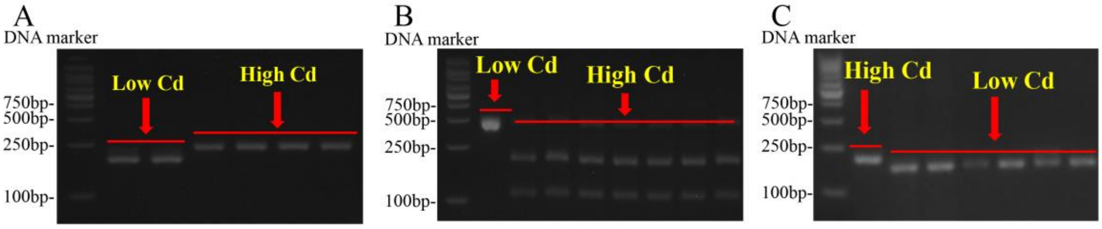

Figure S2. Polymorphism of the three molecular markers between high- and low-Cd accumulating rice cultivars (A: LCd-41; B: LCd-38; C: LCd31).

**Table S2.** Stable low-Cd accumulating early rice cultivars selected based on two field trials **Early rice Cultivars**	**Subspecies**	**Grain Cd concentration (mg kg^{-1})**	
		**Field A**	**Field B**
Zhongguangzao1	*Indica*	0.05	0.04
Zhong'an7	*Indica*	0.06	0.03
Zhong'anzao35	*Indica*	0.10	0.05
Zhongjiazao17	*Indica*	0.18	0.09
Lingliangyou0516	*Indica*	0.18	0.15

Table S3. Genotypes of combined molecular markers for identifying high- and low-Cd accumulating early and late rice cultivars.

	**Combined molecular markers**	**Types**	**Genotypes**
Early rice cultivars	LCd-41-CAL1-LCd-38	Multi-LCL1	AA-GG-CC
		Multi-LCL2	AA-CC-CC
		Multi-LCL3	TT-CC-TT
		Multi-LCL4	TT-CC-CC
Late rice cultivars	CAL1-LCd-31	Multi-CL1	GG-TT
		Multi-CL2	CC-TT
		Multi-CL3	CC-TC
		Multi-CL4	GC-TT
		Multi-CL5	CC-CC

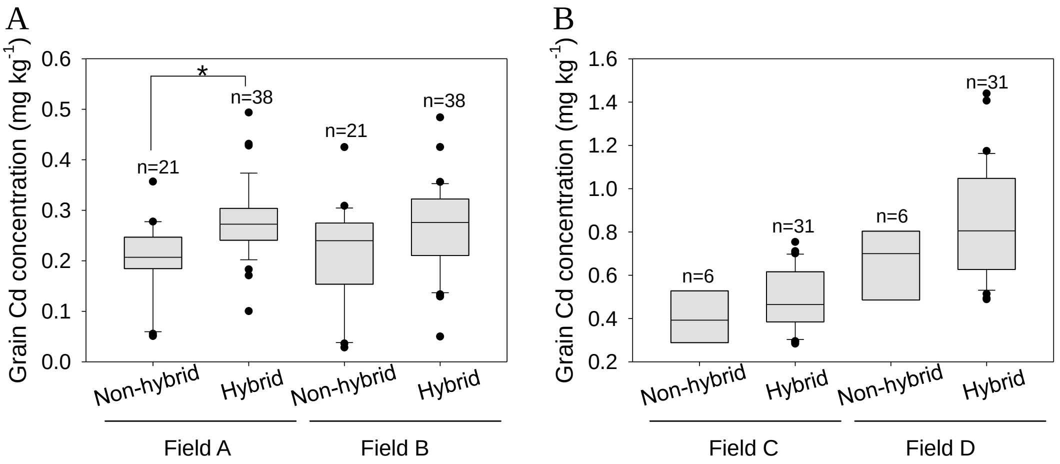

Figure S3. Boxplots of grain Cd concentrations of early (A) and late (B) rice cultivars grouped into hybrid and non-hybrid.

Table S4. Performance of candidate control cultivars 'Zhong'an 2 and 'Shaoxiang 100, demonstrating their unsuitability as stable positive controls due to environmentally variable Cd accumulation and mismatched genotypes.

	**Grain Cd concentration (mg kg^{-1})**		**Combined molecular marker type**
zhong'an2 (early rice)	Field A	0.28	Muiti-LCL4
	Field B	0.04	
Shaoxiang100 (late rice)	Field C	0.38	Muiti-CL2
	Field D	0.03	
